# Size Exclusion HPLC Detection of Small-Size Impurities as a Complementary Means for Quality Analysis of Extracellular Vesicles

**DOI:** 10.5772/61148

**Published:** 2015-07-10

**Authors:** Tao Huang, Anna B. Banizs, Weibin Shi, Alexander L. Klibanov, Jiang He

**Affiliations:** 1 Department of Radiology and Medical Imaging, University of Virginia, Charlottesville, VA, USA; 2 Department of Medicine, University of Virginia, Charlottesville, VA, USA

**Keywords:** extracellular vesicles, exosomes, HPLC, quality analysis

## Abstract

For extracellular vesicle research, whether for biomarker discoveries or therapeutic applications, it is critical to have high-quality samples. Both microscopy and NanoSight Tracking Analysis (NTA) for size distribution have been used to detect large vesicles. However, there is currently no well-established method that is convenient for routine quality analysis of small-size impurities in vesicle samples. In this paper we report a convenient method, called ‘size-exclusion high-performance liquid chromatography’ (SE-HPLC), alongside NTA and Microscopy analysis to guide and qualify the isolation and processing of vesicles. First, the SE-HPLC analysis was used to detect impurities of small-size proteins during the ultra-centrifugation process of vesicle isolation; it was then employed to test the changes of vesicles under different pH conditions or integrity after storage. As SE-HPLC is generally accessible in most institutions, it could be used as a routine means to assist researchers in examining the integrity and quality of extracellular vesicles along with other techniques either during isolation/preparation or for further engineering and storage.

## 1. Introduction

Exosomes and extracellular vesicles are secreted by most cell types under both normal and pathological conditions [1, 2, 3]. These vesicles have been detected in a wide range of biological fluids, such as blood, urine, saliva, and breast milk [4, 5, 6, 7]. Carrying abundant biomolecules like proteins, RNAs and lipids, they are important messengers in intercellular communications and play a pivotal role in tumour progression and metastasis, as well as other diseases [8]. In addition to efforts to understand the biology of exosomes and extracellular vesicles in different diseases, extensive attention has recently also been focused on the study of these nanoparticles as biomarkers and the engineering of vehicles for drug and gene delivery. All such research, which has used different approaches including proteomics [9, 10], [11], transcriptomics [10, 11], lipidomics [10, 12], and vesicle engineering [13, 14], demands samples of high quality.

However, current isolation and characterization methods leave much to be desired in terms of ensuring high-quality vesicles. Various methods have been used for vesicle isolation, including ultracentrifugation [15], size exclusion (filtration or chromatography) [15, 16, 17, 18, 19], immunoaffinity isolation [15, 20], precipitation (ExoQuick or “salting-out”) [21, 22], or combinations of these. Each method yields different results and standardization has not yet been established – though the vesicle research community is working hard on this [23]. A few techniques have been commonly used to characterize isolated vesicles, such as Transmission Electron Microscopy (TEM) or Cryo-EM [24], Nanoparticle Tracking Analysis (NTA, NanoSight) [25], and Western Blot for protein marker confirmation [15]. Recently, Webber et al. proposed the use of the ratio of vesicle counts to protein concentration as an indirect means to check the purity of vesicle preparation [26]. All these approaches are based on the presence of vesicular particles and present some limitations and challenges. For example, electron microscopy was employed to give informative visualization of vesicular morphology and identify the presence of larger-non-vesicular particulates. NTA requires a lot of optimization of parameters and presents several challenges related for example to detection threshold, minimum expected particle size, blur, and minimum track length [25]. On the other hand, for impurity detection, while presence of protein biomarkers not expressed by vesicles [26] was proposed for quality assay, the selection of these markers is challenging and may not be accurate due to the limited understanding of how proteins are packaged into vesicles.

To ensure high-quality vesicles, not only must the desired population of particles be confirmed to be present, contaminants and impurities must also be demonstrated to be absent. Unfortunately, there is no easy method available to rule out the presence of contaminants. Thus, in this paper we report a simple SE-HPLC analysis to detect water-soluble small-size proteins in samples. SE-HPLC is widely used and accessible in most institutions and could be used alongside other current techniques to characterize these exosomes and extracellular vesicles. It could confer a routine and a convenient means to qualify the vesicle products.

## 2. Materials and Methods

### 2.1 Materials

Chemicals or reagents were purchased from commercial sources and used without further processing unless otherwise stated. Research-grade foetal bovine serum (FBS, sterile triple 100 nm filtered) and DMEM cell-culture media were purchased from Fisher Scientific. MDA (MDA-MB-231 human breast adenocarcinoma) and MSTO (MSTO-211H human biphasic mesothelioma) cell lines were from ATCC (Manassas, VA). Sephadex G-25 Nap-5 columns were purchased from GE Healthcare. The ultra-centrifugation was performed on a Beckman Coulter Optima LE-80K ultra-centrifuge with an SW28 rotor. High-performance liquid chromatography (HPLC) was performed on an Agilent 1260 Infinity system equipped with an auto-sampler and a variable wavelength UV detector. The HPLC column was a size-exclusion column (Superose 12 10/300 GL from GE Healthcare) (9 μm-13 μm). The Gel Filtration HMW Calibration Kits were purchased from GE Healthcare and the liposome labelled with DiO (120 nm) for calibration was prepared in-house by Dr Alexander Klibanov. A NanoSight NS300 system (Malvern Instruments Inc., Westborough, MA) and a Tecnai F20 Twin transmission electron microscope (FEI, Hillsboro, OR) were used to characterize the vesicle samples.

## 3. Methods

### 3.1 Cell Culture

Cells, including MDA and MSTO, were cultured at 37°C at 5% CO_2_ in DMEM media complemented with 4% vesicle-depleted FBS. The FBS was depleted of vesicles by overnight ultra-centrifugation at 120,000 g followed by filtration through PVDF 0.22 μm vacuum-driven filters (Millipore). The cell-culture supernatant was collected for vesicle isolation when cell confluence reached 90% (usually after days, from cell seeding to final supernatant collection).

### 3.2 Isolation of Vesicles

**Scheme 1.**
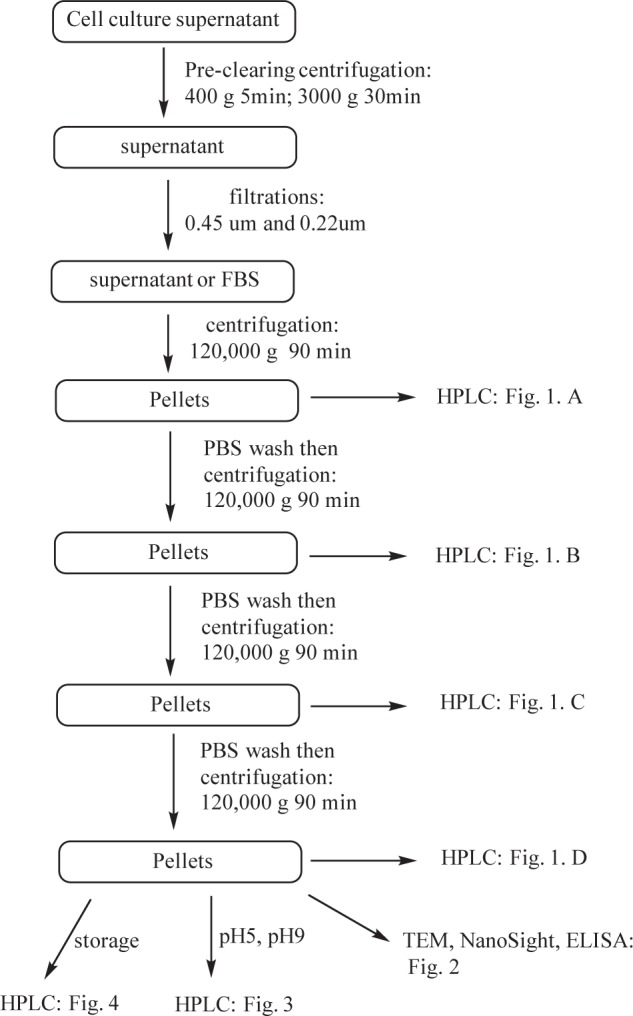
Flow chart of vesicle isolation and purification based on differential ultra- centrifugation combined with filtration. SE-HPLC was used to qualify the intermediates and final product as well.

Vesicles were isolated from cell-culture supernatants or the commercially available FBS. The isolating procedure was adapted from a reported protocol [15] with minor modifications. Briefly, to remove large cell debris the collected cell-culture supernatant was first subjected to subsequent centrifugations at 400 g for 5 min and 3000 g for 30 min, followed by sequential filtration through 0.45 μm and 0.22 μm PVDF filters. FBS was used as purchased. The liquid was further ultra-centrifuged at 120,000 g at 4°C for 90 minutes, followed by removal of the supernatant. The vesicle pellet was subjected to washing by re-suspending in a large volume of fresh DPBS (30 mL) and ultra-centri-fugging at 120,000 g at 4°C for 90 minutes. This washing step was repeated several times and aliquots from each round of ultra-centrifugation were saved for HPLC analysis and other assays, as shown in Scheme 1. Aliquots of final vesicle pellets were used for experiments immediately or stored at 4°C for future use.

### 3.3 Size-exclusion HPLC Analysis

Size-exclusion HPLC (SE-HPLC) was performed with a Superose 12 10/300 GL column with exclusion limit of 2× 10^6^ Daltons to detect the contaminants and vesicular nanoparticles. 50-100 μL of an aliquot was injected via the auto-sampler. The mobile phase was DPBS solution (137 mM NaCl, 2.7 mM KCl, 10 mM Na_2_HPO_4_, 1.8 mM KH_2_PO_4_) with a flow rate of 1.0 ml/min. UV absorbance was detected at a wavelength of 254 nm.

### 3.4 Cryogenic Transmission Electron Microscopy (cryo-TEM) Analysis

Vesicles were vitrified by standard methods of cryo-TEM [27]. In brief, an aliquot (~3.5 μl) was applied to a glow-discharged, perforated carbon-coated grid, manually blotted with filter paper, and rapidly plunged into liquid ethane. The grids were stored in liquid nitrogen, then transferred to a Gatan 626 cryo-specimen holder and maintained at −180°C. Low-dose images were collected at a nominal magnification of 29,000x on a Tecnai F20 Twin transmission electron microscope operating at 120 kV. The digital micrographs were recorded on a Gatan US4000 CCD camera.

### 3.5 NanoSight Particle Analysis

Real-time nanoparticle detection, counting and sizing were performed on the NS-300 NanoSight Instrument following manufacturer protocols. The instrument settings were as follows: camera type – sCMOS; shutter length – varied; shutter setting – 1300; camera gain – 512; frame rate – varied; analysis – blur setting, minimal expected size, minimal track length, all set to automatic. The version of the software was NTA 2.3.

### 3.6 Proteins and ELISA Analyses

Total protein of isolated vesicles and fractions from HPLC were measured using the Micro BCA™ protein assay kit (Thermo Scientific Pierce, Rockford, IL) following the protocol provided by the manufacturer, while the two protein markers, CD63 and CD9, were confirmed by using an enzyme-linked immunoassay kit (ELISA, System Biosciences, Mountain View, CA). All measurements were performed following the protocol provided by the manufacturers.

### 3.7 SE-HPLC Detection of Small-size Impurities in Vesicles at Different pHs or in Refrigerated Storage

The possible changes of vesicles under different pH conditions were evaluated by using SE-HPLC analysis. The buffer medium of purified vesicles was changed from PBS to sodium acetate (0.1M, pH5) or sodium bicarbonate (0.1M, pH9) by using NAP-5 column. Aliquots (about 5×10^8^ particle/mL) were kept at room temperature and analysed by SE-HPLC over time.

To test the possible changes of vesicles in refrigerated storage, an aliquot of vesicles in PBS buffer (pH7.4) was stored at 4°C for up to one month, during which vesicles were analysed weekly by SE-HPLC for possible appearance of small-size impurities, and by Nanosight analysis for the changes of size distribution.

## 4. Results and Discussion

For research seeking to understand the biology of exosomes and extracellular vesicles, to discover biomarkers, especially proteomins, lipidomics and genomics, and to engineer these vesicles for drug and gene delivery, it is crucial to ensure high-quality samples by removing interfering contaminants such as non-vesicular proteins or other biological molecules of small size. To that end, a simple means for quality analysis of vesicle samples is highly desirable. Although several techniques, such as microscopy and NTA, can give visual information on particle size and global size distribution, respectively, there is still no easy technique to detect impurities of small size. Size chromotagraphy has been used for isolation or purification of extracellular vesicles with different gel mediums, but no real HPL analysis has been reported for quality assay (Abstract P-IV-13 at ISEV 2015: Journal of Extracellular Vesicles, 2015,4: 27783). In this paper, we demonstrate that size-exclusion HPLC (SE-HPLC) could be a convenient method to detect proteins and other impurities of small size.

The size-exclusion column used in this study was first calibrated using standard Gel Filtration HMW Calibration Kits (purchased from GE Healthcare) and nano-sized liposomes. The void volume of this column was determined using Blue Dextran 2000 (molecular weight 2000 kDa) to be at 8 min and 4 sec at a flow rate of 1 mL/min. Further confirmed by DiO-labelled liposomes (120 nm), components eluting before 8 min were not resolved since they were within the void volume. Proteins as big as Thyroglobumin and Ferritin can be resolved on this column (see details in supporting materials), so it is selected to separate non-associated proteins and other smaller impurities from vesicles.

**Figure 1.**
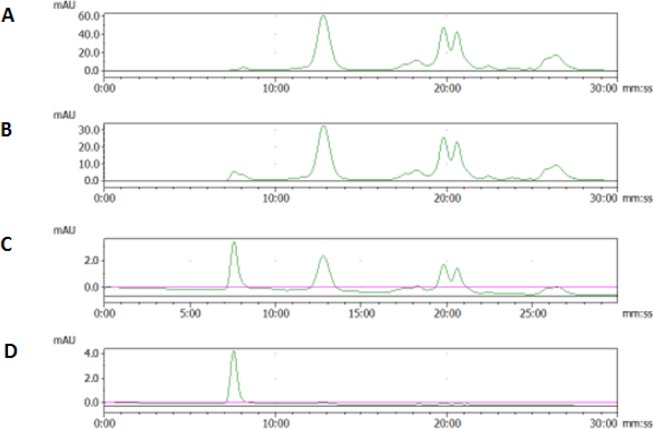
Representative chromatograms of extracellular vesicles from MSTO cell-culture supernatant. Aliquots after one (A), two (B), three (C) and four (D) ultra-centrifugations were analysed. The peak with a retention time of 7.5 min stands for the exosome particles while the other peaks are impurities of small size.

**Figure 2.**
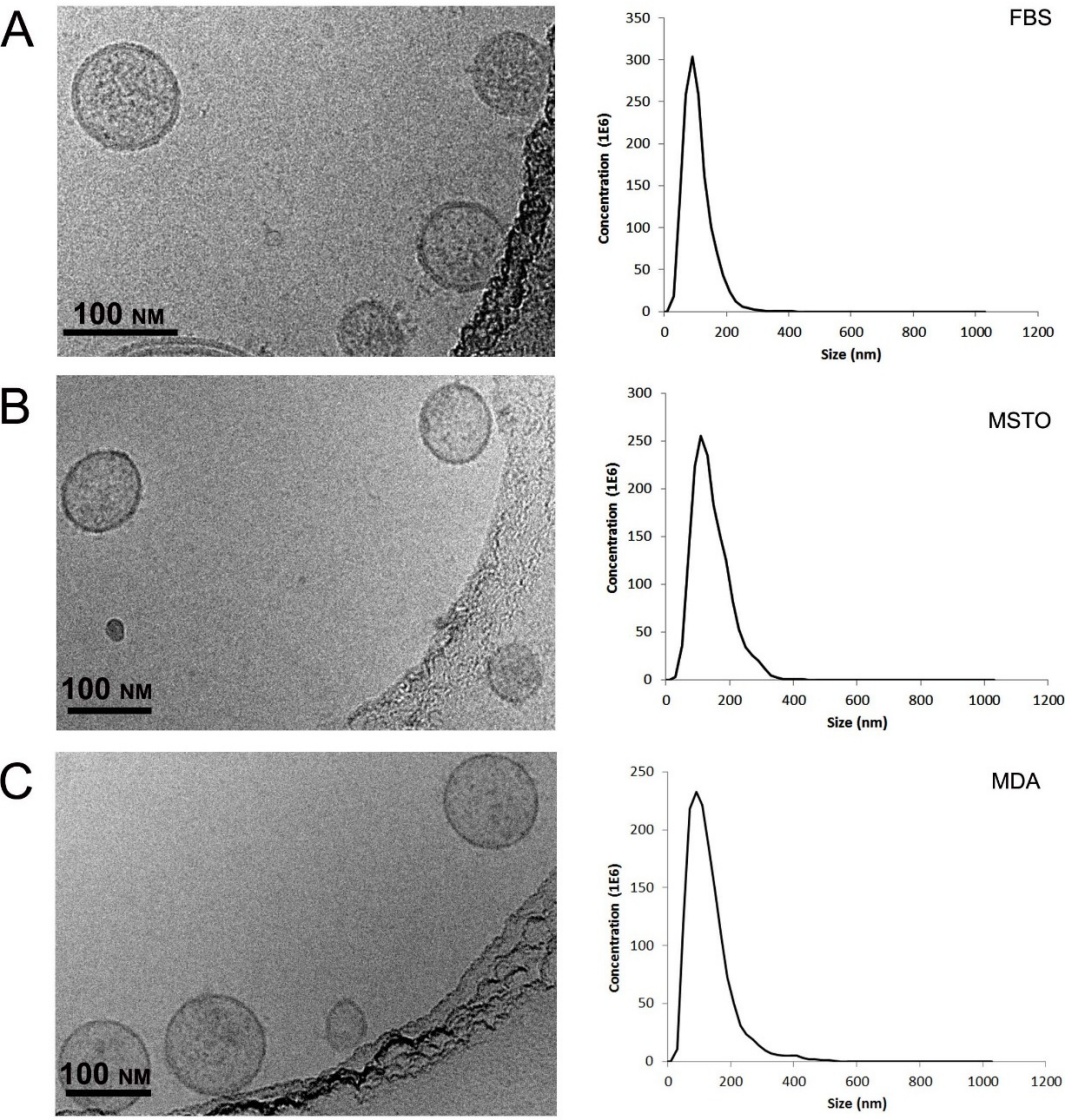
Characterization of purified extracellular vesicles isolated from FBS (**A**), MSTO cells (**B**), and MDA cells (**C**). Representative Cryogenic Transmission Electron images of vesicles show round-shape membrane-bound particles from all three sources (FBS, MSTO and MDA cells; left column) with occasional irregularly distributed electron-dense content, both inside and on the surface of the membrane. The NanoSight plots depict typical size distributions of extracellular vesicles from the above-described sources (right column). Bars correspond to 100 nm.

Several techniques have been used to isolate vesicles. In this study, sequential ultra-centrifugation was employed to prepare vesicles from MSTO, MDA tumour cells and FBS, and the samples were analysed by SE-HPLC. According to the ultra-centrifugation protocol [15], three rounds of ultra-centrifugations (70 min each run) were used to isolate and purify vesicles from cell-conditioned media. Since the procedure is empirical, the reproducibility of high-quality vesicles remains an issue. By introducing SE-HPLC to separate and detect vesicles and free small-size proteins, we were able to analyse the aliquot at different centrifuging levels in the whole process. As demonstrated in Fig. 1, after two rounds of ultra-centrifugation, the species eluting out of the column before 10 min were negligible in the mixture compared to those appearing after 10 min. After the third round of ultra-centrifugation, the peak with the retention time of 7.5 min (identified as vesicle peak as shown in Fig 2) became more dominant while other peaks after 10 min were decreased. With one more round of washing and ultra-centrifugation, a single peak was observed at 7.5 min, which represented the final vesicle product. All three kinds of vesicles from different sources (FBS, MSTO, MDA) showed similar patterns in HPLC analysis for this ultra-centrifugation process.

The vesicle samples representing the single peak in SE-HPLC were subjected to cryo-TEM and NanoSight analysis (Fig. 2). In TEM, these vesicles appeared as well-defined membrane-bound vesicles ranging in size from 30 to 150 nm in all three kinds of vesicle. NanoSight analysis gave a more broad distribution of 40 to 200 nm. To further confirm the identity of the samples representing the different peaks in SE-HPLC, fractions at 7.5 min and 12.8 min were collected and examined by cryo-TEM and ELISA for the presence of extracellular protein markers CD63 and CD9. The results showed that the fraction at 7.5 min was extracellular-vesicles positive in both CD63 and CD9, while the fraction sample at 12.8 min was not positive in either of these.

**Figure 3.**
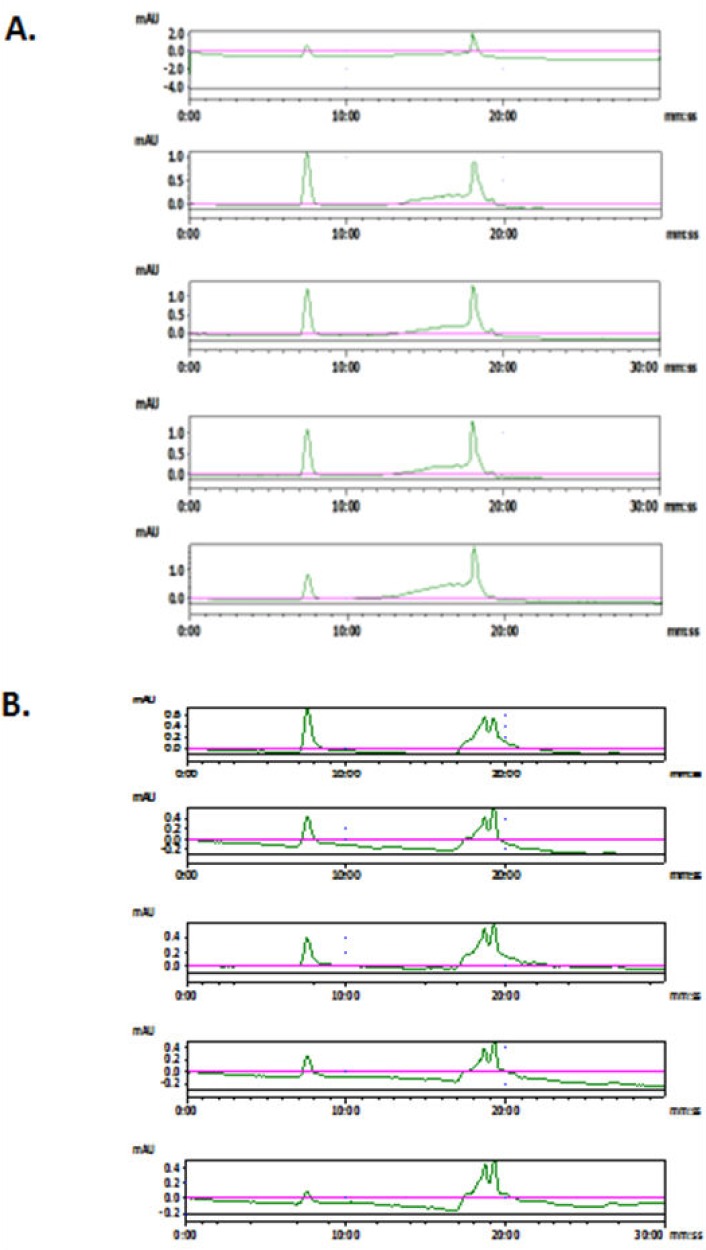
Chromatograms of FBS vesicles in (**A**) bicarbonate buffer (0.1M, pH9) and (**B**) acetate buffer (0.1M, pH5). From top to bottom in both A and B, the time point was 0h, 1h, 2h, 4h and overnight (14h), respectively.

In recent years, nano-sized extracellular vesicles have emerged as potential drug- and gene-delivery carriers, where nano-sized vesicles may need to be subjected to pH conditions other than the physiological one (pH 7.2-7.4). To test for any changes in vesicles under different pH conditions, the buffer for vesicle samples was changed from PBS (pH7.4) to sodium acetate buffer (pH5) or sodium bicarbonate buffer (pH9). The SE-HPLC analyses were performed at different times. As illustrated in Fig. 3, FBS vesicles showed a response to pH changes, with intensity of vesicle peak continuously decreasing while that of newly generated peaks increased in the study period. Although the details of these new peaks are not known, and further identification is challenging and beyond the focus of the current study, it is apparent that changes did happen in these vesicles. These new impurity peaks might be due to the vesicular particles disrupted or the proteins or other biomolecules of small size falling off from the vesicles, resulting the lower and lower signal from the vesicles. Nevertheless, we have demonstrated that it would be useful to use SE-HPLC to detect impurities or changes of vesicles under different conditions.

**Figure 4.**
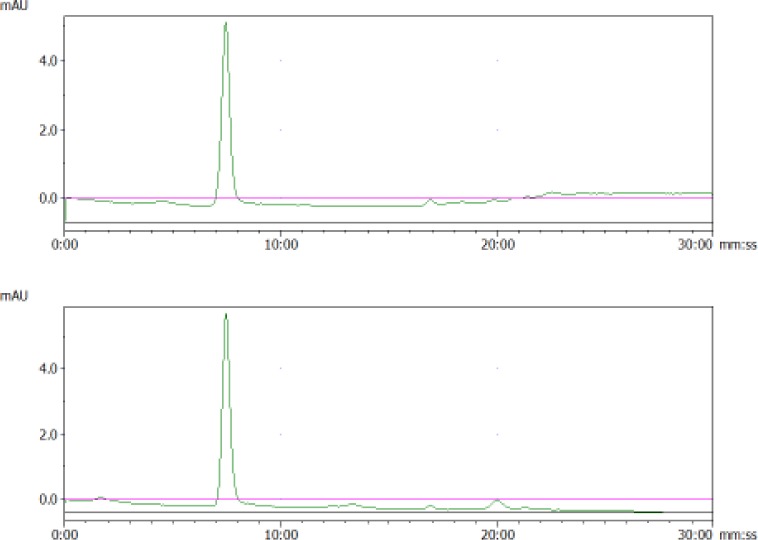
Representative chromatograms of MDA vesicles stored at 4°C for one month. The top was fresh aliquot and the bottom was aged for one month in a 4°C refrigerator.

Another question for vesicle research regards the appropriate storage condition for vesicle samples. Generally, −80°C has been recommended [23] for long-term storage. However, the cycle of “freeze and thaw”, if it happens frequently, may do harm to membrane vesicles. Is it safe to keep the vesicles at 4°C, and how long will they remain good? As illustrated in Fig. 4, aliquots of purified MDA vesicles in PBS were kept at 4°C for up to one month and there was no obvious change in the SE-HPLC graph. The NTA analysis also showed minimum changes of size in these samples. The other two vesicle samples from MSTO cells and FBS showed similar results, without any substantial changes in size distribution and small-size impurities. These results suggest that it may be safe to keep vesicle aliquots in a refrigerator for a short period of up to four weeks. However, our SE-HPLC evidence only implies physical stability as particulates at 4°C. To draw conclusions about the biological functions of these extracellular vesicles after storage, further evaluation is required.

It is important to note that there are also several limitations related to this HPLC detection technique. First, in our study, no detailed information on vesicle size is gained by HPLC analysis alone, since this column cannot resolve nanoparticles of different size. All nanoparticles elute at the void volume as demonstrated by calibrations where Blue Dextran 2000 (2, 000 k Daltons) and DiO-labelled liposomes of size 120 nm were used to determine the void volume under the same condition used for vesicle samples (shown in supporting materials). The intention of this study was to test whether SE-HPLC can be used as a means of quality analysis of soluble proteins and other impurities of small size. For a detailed size measurement by HPLC, more evaluations are needed. It would be best to use the simple and straightforward SE-HPLC to qualify the vesicle preparation followed by other characterizations such as TEM, NTA, western blot and flow cytometry. Secondly, a high exclusion limit of 2×10^6^ Daltons was used in this study to give a wide detection range and better resolution of small impurities for the extracellular vesicles of size less than around 200 nm. Other exclusion limit ranges may give different results, depending on the vesicles and the sources to prepare these vesicles. From our experience, size-exclusion columns with higher exclusion limits may be able to resolve large particles but will give less desirable resolution of small-size molecules. Therefore, it is important to choose an appropriate column and calibrate it with a series of molecules of different sizes. Finally, these results were based on the ultra-centrifugation purification method using commercially available FBS and tumour cells. For biomarker discovery research dealing with real samples of blood, etc., different isolation techniques such as affinity selection, filtration, or others, as well as any combinations of these could be employed to generate particular types of vesicles. Nonetheless, purity of samples would be the top priority to avoid false biomarkers due to impurities. Therefore, in addition to quality-analysis techniques (NTA as a global analysis of size distribution) to exclude large particles or aggregates, techniques such as the HPLC analysis reported here represent a convenient complementary means to detect impurities of small size.

## 5. Conclusions

In this study, we conclude that size-exclusion HPLC analysis can be used to help determine the purity of extracellular vesicle samples by detecting proteins and other impurities of small size that can have a substantial impact on biomarker discovery. This method has been used to examine the purity of vesicular nanoparticles with changes in pH and under storage at 4°C. The results exemplify the potential of such HPLC analysis to monitor vesicle disruption during storage, modification or other processes in extracellular vesicle research focusing on biomarker discovery or therapeutic application.

## 6. Compliance with Ethical Research Standards

The authors declare no conflicts of interest. No part of this study was performed on any human or animal subjects.
